# Mechanical properties of γ-graphyne nanotubes

**DOI:** 10.1039/c8ra01970c

**Published:** 2018-04-25

**Authors:** Maoyuan Li, Yingming Zhang, Yunliang Jiang, Yun Zhang, Yunming Wang, Huamin Zhou

**Affiliations:** State Key Laboratory of Material Processing and Die & Mold Technology, Huazhong University of Science and Technology Wuhan 430074 Hubei China wang653@hust.edu.cn +86-27-87543492; Key Laboratory for Material Chemistry of Energy Conversion and Storage, Ministry of Education, School of Chemistry and Chemical Engineering, Huazhong University of Science and Technology Wuhan 430074 Hubei China

## Abstract

γ-Graphyne nanotubes (γ-GNTs), which are formed by rolling up a γ-graphyne sheet in a similar way to carbon nanotubes, exhibit unique mechanical properties due to the carbon atoms in the sp and sp^2^ hybridized states. In this study, the mechanical properties of γ-GNTs were investigated using molecular dynamics simulations. The effects of the dimensions, temperature, strain rate and the presence of a vacancy on the mechanical properties, *i.e.*, Young’s modulus, fracture strength and fracture strain, were comprehensively studied. The results indicate that the mechanical properties of the γ-GNTs are not sensitive to the length and strain rate, while the Young’s modulus increases with increasing diameter. Meanwhile, an obvious temperature-dependent mechanical behavior was also found due to the stronger thermal vibration of the atoms at a higher temperature, especially in terms of the fracture strength and fracture strain. In addition, the mechanical properties of the γ-GNTs would be degraded with the existence of a vacancy, and they are more sensitive to the vacancy in the benzene rings than that in the acetylenic linkages, especially for the double-vacancy. The underlying mechanisms were analyzed from the stress distribution and fracture structure during tensile deformation.

## Abstract 2

The effects of the dimensions, temperature, strain rate and the presence of a vacancy on the mechanical properties of γ-GNTs were systematically investigated using MD simulations.

## Graphical abstract

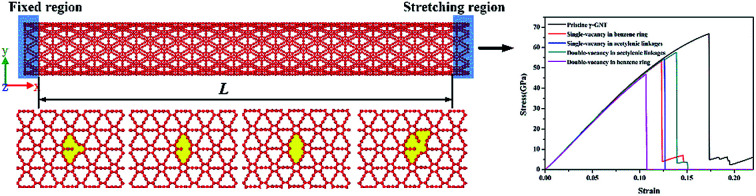

## 1. Introduction

In recent years, graphene^1^ and its allotropes, such as graphyne^2^ and T-carbon,^3^ have gained tremendous attention due to their unusual mechanical, thermal and optical properties.^2–8^ First predicted by Baughman *et al.*,^9^ graphyne has a two dimensional structure, formed in the sp and sp^2^ hybridized states, which is different from graphene, containing sp^2^ bonds only. This novel carbon allotrope exhibits a low density, a great capability for water desalination and unique mechanical and thermal properties.^2,10–14^ Since the fabrication and characterization of graphyne are difficult and complex, simulation methods, including molecular dynamics (MD) simulations, the density functional theory (DFT) method and the *ab initio* molecular dynamics (AIMD) method, are widely used for exploring the different properties of graphyne. For example, Sarkar *et al.*^7,15,16^ conducted a series of investigations on the electronic, optical and other physical properties of pristine/doped graphyne nanotubes using the DFT method. Cranford *et al.*^5^ found that the fracture behavior of γ-graphyne exhibited strong anisotropy, which was related to the direction of applied strain and the alignment of acetylenic linkages. Zhang *et al.*^17^ demonstrated that the Young’s modulus and fracture strength of γ-graphyne were much lower than those of graphene, and this can be attributed to the lower atom density and weaker single bonds in the acetylenic linkages in γ-graphyne. They also found that the thermal conductivity of γ-graphyne was significantly suppressed due to the low atom density caused by the acetylenic linkages in the structures.

From the view of topology, γ-graphyne nanotubes (γ-GNTs) can be formed by rolling up γ-graphyne sheets into seamless cylinders, in a similar way to carbon nanotubes (CNTs). The mechanical and thermal stabilities of γ-GNTs have been confirmed by theoretical calculations and show some different properties when compared with CNTs. For instance, Coluci *et al.*^18^ indicated that the band gap values of γ-GNTs were independent of the tube diameter or chirality. Hu *et al.*^19^ showed that γ-GNTs exhibit an unprecedented low lattice thermal conductivity resulting from the larger vibrational mismatch between the weak acetylenic linkages (sp carbon bonds) and the strong benzene ring (sp^2^ carbon bonds). More recently, Sousa *et al.*^20^ found that γ-GNTs exhibit “superplasticity” behavior, as compared with CNTs, which is due to the irreversible reconstruction process during torsional strain with the existence of acetylenic linkages.

Although there have been some pioneering simulation studies on the electronic and thermal properties of γ-GNTs, as described above, there have been few investigations on the mechanical properties of γ-GNTs. To our knowledge, a comprehensive study on the mechanical properties of γ-GNTs is still lacking. The mechanical properties of γ-GNTs are of great significance as γ-GNTs may also have the potential to be applied as a reinforcing nanofiller in some nanocomposites, in a similar way to CNTs. Earlier studies^21^ found that γ-graphyne sheets exhibit temperature and strain rate-dependent mechanical behavior. Meanwhile, the Young’s modulus, fracture strength and strain of γ-graphyne sheets would observably decrease with the existence of a vacancy. Inspired by this work, it is of great interest to investigate the mechanical properties of γ-GNTs with different tube diameters, temperatures, strain rates and defects.

Therefore, using MD simulations, the mechanical properties of γ-GNTs were comprehensively investigated in this paper. The effects of tube diameter, length, temperature, strain rate and the presence of a vacancy were explored. The Young’s modulus, fracture strength and strain were obtained and discussed. Moreover, the fracture mechanisms of the γ-GNTs were also analyzed from the stress distribution and fracture structure during tensile deformation.

## 2. Computational methods

**Fig. 1:**
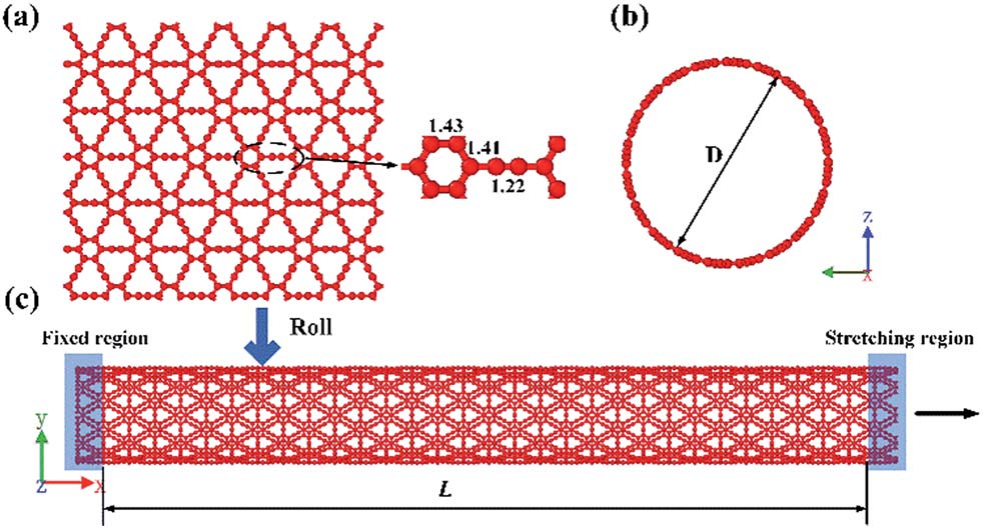
A schematic diagram of: (a) a γ-graphyne sheet and (b) the lateral view and (c) the front view of a γ-GNT. The arrow indicates that uniform strain was applied along the *x*-axis direction.

The atomic structure of a γ-GNT is shown in Fig. 1(c), and it is formed by rolling up a γ-graphyne sheet (as shown in Fig. 1(a)). The optimized bond length values were chosen according to previous density functional theory calculations.^22^ The chirality-dependent effect on the mechanical properties of γ-GNTs has been comprehensively investigated by Wu *et al.*^23^ Their results indicated that the fracture strength and strain of γ-GNTs in the zigzag direction are higher than those in the armchair direction. Our work is mainly focused on the effects of the dimensions, temperature and defects on γ-GNTs with zigzag chirality. γ-GNTs, with a diameter of 19.77 Å and a length of 96.61 Å, were used without special treatment.

In the MD simulations, the adaptive intermolecular reactive bond order (AIREBO) potential^24^ was used to describe the C–C bonding interactions. This force field has been successfully applied in previous studies to investigate the mechanical/thermal properties of carbon-based systems, such as graphene and graphynes.^5,21,25–27^ The AIREBO potential can be expressed as:1
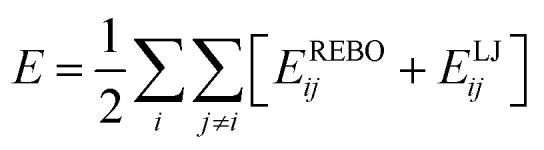
where *E*^REBO^_*ij*_ and *E*^LJ^_*ij*_ indicate the short-range C–C interactions (*r* < 2.0 Å) and long-range interactions (2.0 Å < *r* < cut-off distance), respectively. The cutoff function for the C–C bond distance in the AIREBO potential was increased from 1.7 Å to 2.0 Å to avoid unphysical results before fracture occurs, which has been validated by previous studies.^21,28^ The total potential energy of the initial system was first minimized using a conjugate gradient algorithm. The system was then relaxed in a canonical NVT ensemble (*i.e.*, a constant number of atoms, volume and temperature) at different temperatures for 100 ps. Uniaxial tension simulations were conducted, followed by equilibration, to determine the mechanical properties of the γ-GNTs. The atoms near both ends of the γ-GNT were fixed, and the length of the two fixed regions was set at about 5 Å, as shown in Fig. 1(c). A constant uniaxial strain was applied to the right end along the *x*-direction with a strain rate. A relaxation time of 10 ps in the NVT ensemble was applied to ensure the system was fully equilibrated after every step of tension. Nonperiodic boundary conditions were applied in the three directions. The MD simulations were conducted at a temperature of 1 K and a strain rate of 1.0 × 10^−4^ ps^−1^ unless otherwise stated. A time step of 0.5 fs was used in all of the simulations. All of the MD simulations were conducted using the Large-scale Atomic/Molecular Massively Parallel Simulator (LAMMPS),^29^ and the velocity-Verlet method was used to integrate the equation of motion.

The atomic stress of the γ-GNT during uniaxial tension was calculated using the viral theorem and the following equation:^30^2
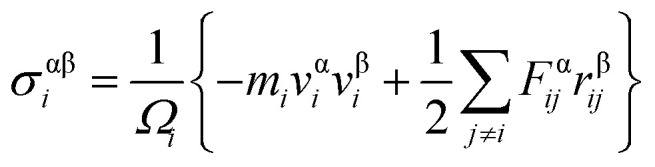
where *Ω*_*i*_, *m*_*i*_ and *v*_*i*_ represent the volume, mass and velocity of an atom *i*, respectively. *F*_*ij*_ and *r*_*ij*_ are the force and distance between atoms *i* and *j*, respectively, and the indices α and β denote the Cartesian coordinate components. According to previous studies,^5,23^ a thickness of 3.2 Å for the γ-GNT was used to calculate the effective atomic volume. By averaging over the atomic stresses of all of the atoms, the mechanical properties of the γ-GNTs, *i.e.*, Young’s modulus, fracture strength and fracture strain, could be obtained from the analysis of the stress–strain curves.

## 3. Results and discussion

### 3.1 Effect of diameter and length

**Fig. 2:**
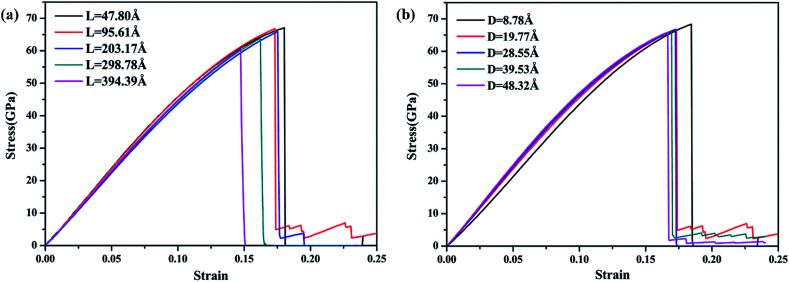
Stress–strain curves for the γ-GNTs at different (a) lengths and (b) diameters.

To validate the modeling and to assess the generality of the mechanical characteristics of the γ-GNTs, the effects of diameter and length on the mechanical properties were first investigated. The length of the γ-GNT was defined as the effective length during stretching, as shown in Fig. 1(c). The diameter and length values for the γ-GNTs ranged from 8.78 to 48.32 Å and 47.80 to 394.39 Å, respectively. As shown in Fig. 2, the stress–strain curves indicate that the stress increases with the strain until breaking and there is no evident plastic deformation period, which is similar to that observed for γ-graphyne sheets.^21^ The Young’s modulus was calculated by linear fitting of the stress–strain curves when the strain was <2%, *i.e.* at elastic deformation. The value of the fracture strength was defined as the maximum stress, and the corresponding strain was the fracture strain.

**Fig. 3:**
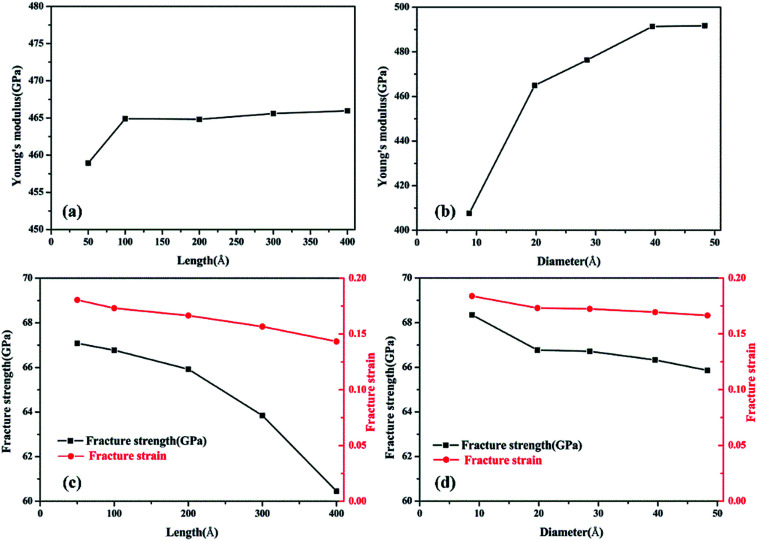
The fracture strength of γ-GNTs with different (a) lengths and (b) diameters; the fracture strain of γ-GNTs with different (c) lengths and (d) diameters.

**Fig. 4:**
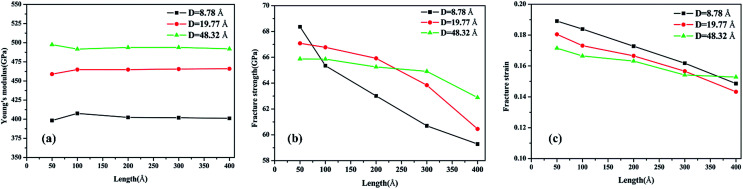
Variation of the (a) Young’s modulus, (b) fracture strength and (c) fracture strain with the tube length at different diameters.

The corresponding Young’s modulus, fracture strength and fracture strain were calculated and the results are displayed in Fig. 3. When the value of the diameter was fixed (*i.e.*, 19.77 Å in this study), the Young’s modulus slightly increased with the increase in length, and then reached a plateau at ∼465 GPa (the length was ∼100 Å). This value is much smaller than that observed for CNTs,^31^*i.e.*, ∼935 GPa, and this is due to the existence of acetylenic linkages in γ-GNTs. However, the fracture strength and strain decreased with the increase in length, indicating that γ-GNTs with shorter lengths are more stable than those with longer lengths. When the length increased from 47.80 to 394.39 Å, the fracture strength decreased from 67.08 to 60.45 GPa, and the fracture strain decreased from 0.180 to 0.143. Such a phenomenon is consistent with that observed for σ-graphyne^32^ and CNTs.^33^ In addition, the Young’s modulus, fracture strength and fracture strain of the γ-GNTs with different diameters (8.78 to 48.32 Å) were also calculated, as shown in Fig. 4. The results show that the Young’s modulus increased slightly with the increase in length, and then reached a plateau for the γ-GNTs with different diameters. However, both the fracture strength and strain are more sensitive to length changes in the low diameter tubes, *e.g.* the fracture strength decreased by 4.55% (from 65.88 to 62.88 GPa) for a diameter of 48.32 Å, and it decreased by 13.3% (from 68.36 to 59.29 GPa) for a diameter of 8.78 Å. Such behavior can be attributed to the fact that the mixing of σ and π orbitals changes rapidly, especially for γ-GNTs with a low diameter.^6^

When the value of the length was fixed (*i.e.*, 95.61 Å in this study), the results show that the Young’s modulus increased remarkably with the increase in diameter, while both the fracture strength and strain slightly decreased. The value of the Young’s modulus increased from 407.60 to 491.67 GPa when the diameter increased from 8.78 to 48.32 Å. Moreover, the Young’s modulus reached saturation when the diameter was larger than a critical value, *i.e.*, 39.53 Å in this study. This trend is similar to the results for tube-like materials, such as CNTs^34^ and single/double silicon carbide nanotubes.^4,35^ In previous work by Chang *et al.*,^34^ a closed-form expression for the Young’s modulus as a function of the nanotube diameter was presented based on the molecular mechanics method, and the obtained results indicate that the Young’s modulus would increase with the diameter and reach a plateau. Meanwhile, the results also clearly show that the effect of diameter is more significant than that of the length on the Young’s modulus of γ-GNTs. Such a phenomenon could be explained on the basis of σ and π orbital mixing. As stated in ref. 6, the orthogonal relation between the σ and π orbitals no longer exists when the graphyne nanosheet is rolled up to form a tube. The σ and π orbital mixing could have a significant influence on the mechanical properties, while the influence becomes larger as a result of the greater curvature and strain for low diameters.

### 3.2 Effect of temperature and strain rate

**Fig. 5:**
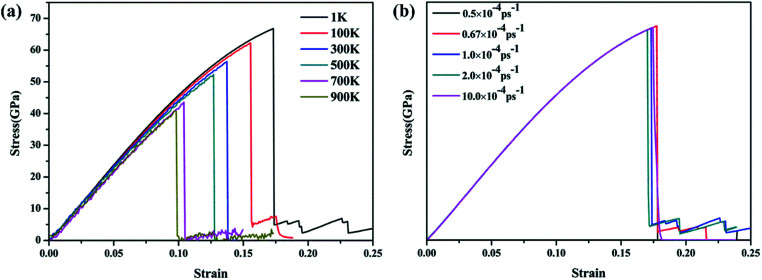
Stress–strain curves for γ-GNTs at different (a) temperatures and (b) strain rates.

The temperature- and strain rate-dependent effects are of great importance for the mechanical properties of low-dimensional materials, thus the effects of temperature and strain rate were investigated. The stress–strain curves of the γ-GNTs at different temperatures (from 1 to 900 K) and strain rates (from 0.5 × 10^−4^ to 10 × 10^−4^ ps^−1^) were obtained and are displayed in Fig. 5(a) and (b), respectively. The curves at different temperatures and strain rates indicate that all of the γ-GNTs exhibit similar deformation behavior.

**Fig. 6:**
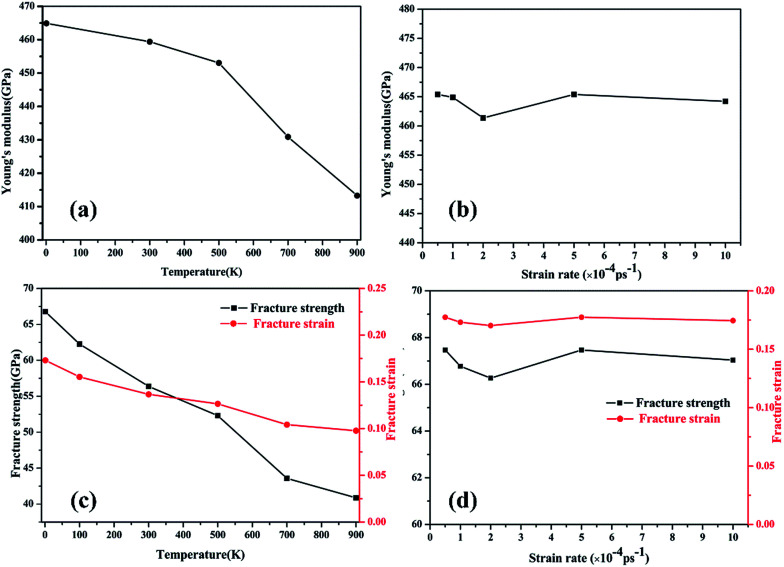
The fracture strength of γ-GNTs at different (a) temperatures and (b) strain rates; the fracture strain of γ-GNTs at different (c) temperatures and (d) strain rates.

**Fig. 7:**
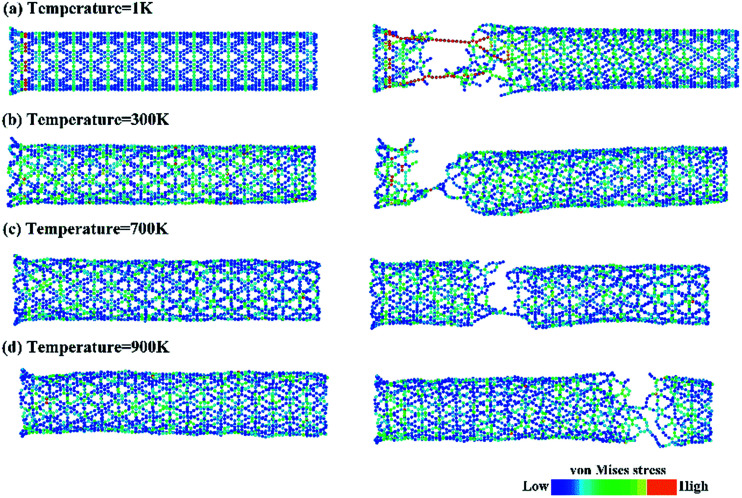
A snapshot of the deformation process and the von Mises stress distribution for γ-GNTs at temperatures of (a) 1 K, (b) 300 K, (c) 700 K and (d) 900 K.

The corresponding Young’s modulus, and fracture strength and strain at different temperatures and strain rates are shown in Fig. 6. With regard to temperature, the results indicate that the values of the Young’s modulus, and fracture strength and strain decreased with the increase in temperature. When the temperature was increased from 1 to 900 K, the Young’s modulus decreased from 464.91 to 413.27 GPa, the fracture strength decreased from 66.77 to 40.86 GPa, and the fracture strain decreased from 0.173 to 0.097. Such temperature-dependent mechanical behavior is consistent with previous studies on γ-graphyne^21^ and CNTs,^36^ and can be attributed to the stronger thermal vibration of atoms at a higher temperature.^37^ Since the breaking of the carbon–carbon bonds (including single, double and triple-bonds) is more likely, γ-GNTs are less stiff at higher temperatures. Moreover, snapshots of the deformation process and the von Mises stress distribution at different temperatures are shown in Fig. 7. The formation of carbon chains can be observed ranging from 1 to 900 K, indicating that the γ-GNTs exhibit classical ductile characteristics. Similar behavior was also reported for CNTs by Heine *et al.*^38^ Their results showed that the zigzag CNTs exhibited more brittle properties at ambient temperature, while they behaved as ductile materials at higher temperature. Such a difference is mainly due to the existence of acetylenic linkages in γ-GNTs, which are more likely to form a chain when compared with the benzene rings of CNTs.

With regard to the strain rate, the stress–strain curves at different strain rates coincide with each other before the fracture occurs, revealing that the strain rates have a negligible effect on the Young’s modulus. Meanwhile, the fracture strength and strain are slightly decreased at a lower strain rate. This strain rate-dependent behavior is due to the fact that there is more time for the thermal vibration of atoms at a lower strain rate, leading to more possibility for C–C bond breaking.^21,37^ However, the effect of strain rate on the mechanical properties is less significant than the temperature effect.

### 3.3 Effect of single/double-vacancy

**Fig. 8:**
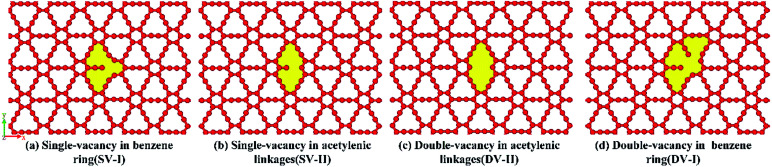
The types of defects studied in this work: single-vacancy in (a) a benzene ring (SV-I) and (b) an acetylenic linkage (SV-II); double-vacancy in (c) an acetylenic linkage (DV-II) and (d) a benzene ring (DV-I).

**Fig. 9:**
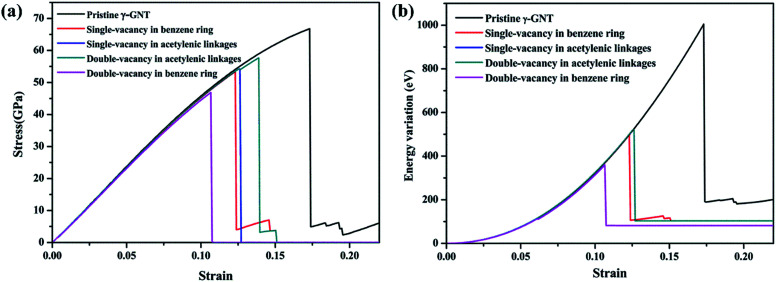
(a) Stress–strain curves for γ-GNTs and (b) total energy variations during the loading process for different types of vacancy defect. The temperature was set at 1 K.

Finally, we have examined the single- and double-vacancy effect on the mechanical properties of γ-GNTs. Four types of vacancy in the γ-GNTs are considered, *i.e.*, the single-vacancy in the benzene ring (SV-I), the single-vacancy in the acetylenic linkages (SV-II), the double-vacancy in the acetylenic linkages (DV-II) and the double-vacancy in the benzene ring (DV-I), as shown in Fig. 8. To reduce the impact of other factors, the position of the vacancy is set in the middle of the γ-GNT for the above defects, and only one defect is created. As shown in Fig. 9(a), the stress–strain curves for the γ-GNTs with/without the four types of vacancy show similar elastic deformation behavior before fracture occurs. The total energy variations during the loading process were obtained and are displayed in Fig. 9(b), and the results also indicate that it requires more energy to break the pristine γ-GNT when compared with the γ-GNTs with a vacancy.

Based on the stress–strain curves, the Young’s modulus, fracture strength and fracture strain of the γ-GNTs with/without a vacancy are presented in Table 1. To evaluate the temperature-sensitive effects for the different vacancies, temperatures of 1 K and 700 K were considered. The results clearly show that the Young’s modulus, fracture strength and fracture strain will be remarkably reduced with the introduction of a vacancy. The single-vacancy in the benzene ring shows a similar effect on the mechanical properties to the single-vacancy in the acetylenic linkages, while the effect of the double-vacancy in the benzene ring on the mechanical properties is more significant than that of the double-vacancy in the acetylenic linkages, especially for the fracture strength and strain. For example, the double-vacancy in the benzene ring causes a 29.8% (from 66.77 to 46.88 GPa) reduction in the fracture strength and a 38.7% (from 0.173 to 0.106) reduction in the fracture strain, while the values are around 13.6% (from 66.77 to 57.67 GPa) and 19.6% (from 0.173 to 0.139) for the double-vacancy in the acetylenic linkage. This could be attributed to the ultrahigh stiffness of the γ-GNT, which is mainly due to the effect of the benzene rings. Previous studies^21^ have also shown that the fracture strength and strain decrease with the increasing percentage of acetylenic linkages. Meanwhile, the reduction in the fracture strength and strain due to increasing temperature is 12.8% and 9.4%, respectively, for the double-vacancy in the benzene ring, while the values are 36.6% and 36.7% for the double-vacancy in the acetylenic linkages.

**Table 1:** The Young’s modulus, fracture strength and fracture strain of the γ-GNTs with/without a vacancy at temperatures of 1 and 700 K

Model	Temperature	Young’s modulus (GPa)	Fracture strength (GPa)	Fracture strain
Pristine γ-GNT	1 K	465.49	66.77	0.173
	700 K	430.87	43.57	0.104
Difference		7.4%	34.7%	39.9%
SV-I	1 K	457.49	53.37	0.123
	700 K	430.00	35.91	0.105
Difference		6%	32.7%	14.6%
SV-II	1 K	457.59	54.26	0.126
	700 K	427.46	37.73	0.092
Difference		6.6%	30.5%	27.0%
DV-II	1 K	457.83	57.67	0.139
	700 K	441.08	36.59	0.088
Difference		3.7%	36.6%	36.7%
DV-I	1 K	449.78	46.88	0.106
	700 K	441.53	40.86	0.096
Difference		1.8%	12.8%	9.4%

**Fig. 10:**
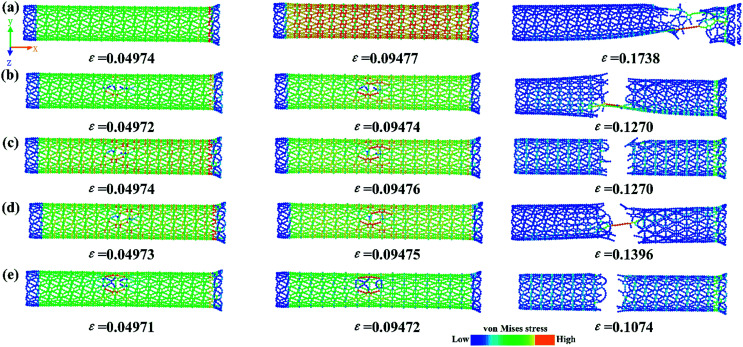
A snapshot of the deformation process and von Mises stress distribution at different strains for (a) pristine γ-GNT, (b) SV-I, (c) SV-II, (d) DV-II and (e) DV-I.

To gain a deeper insight into the underlying mechanisms of the effect of a vacancy, we further investigated the stress distribution and fracture behavior during tensile deformation. The von Mises stress distributions at different strains for the γ-GNTs with/without a vacancy are shown in Fig. 10. For the pristine γ-GNT, the stress distribution was uniform during the initial stage (*ε* = 0.04874) and the atoms in the tensile direction (*x*-axial) showed a higher stress than those in the vertical direction (*y*-axial) during further deformation (*ε* = 0.09477). Since the C–C bonds in the benzene ring are stronger than those in the acetylenic linkages, bond breaking will occur at the acetylenic linkages and the fracture starts near the boundaries due to boundary effects (*ε* = 0.1738), which is similar to that observed for γ-graphyne sheets.^17,39^ For the γ-GNT with a vacancy, a clear stress concentration distribution occurs near the vacancy, indicating that the atoms near the vacancy bear a higher stress. Herein, the fracture starts at vacancy locations and propagates perpendicularly to the loading direction. The fracture structures of these simulations are also consistent with the characteristics of the stress–strain curves, as shown in Fig. 9(a).

## 4. Conclusion

In summary, the mechanical properties of γ-GNTs have been investigated through a series of MD simulations. The effects of the system dimensions were first studied, and the results indicate that the Young’s modulus increased with the diameter and was independent of the length, while both the fracture strength and fracture strain slightly decreased with the increasing diameter and length. Meanwhile, the temperature- and strain rate-dependent mechanical behaviors were also investigated. The simulation results show that the Young’s modulus, fracture strength and fracture strain all decreased with increasing temperature, while these values changed slightly with the strain rates.

In addition, the effect of a vacancy on the mechanical properties was investigated. Four types of vacancy, *i.e.*, a single/double-vacancy in the benzene ring and a single/double-vacancy in the acetylenic linkages, were considered. It was found that the mechanical properties of the γ-GNTs would be degraded with the existence of a vacancy and the vacancy in the benzene ring showed a stronger effect when compared with that in the acetylenic linkages. The underlying mechanism of the vacancy effect was analyzed from the stress distribution and fracture structure during tensile deformation. The above findings provide a comprehensive understanding of the mechanical properties and fracture mechanism of γ-GNTs. Furthermore, the present study could shed light on the outstanding importance of the dimensions, temperature and vacancy effects on the mechanical properties of γ-GNTs, which provides useful guidance for the design and application of graphyne-based nanodevices.

## Conflicts of interest

The authors declare no competing financial interest.

## Supplementary Material
